# Accuracy and limitation of plaque detection by coronary CTA: a section-to-section comparison with optical coherence tomography

**DOI:** 10.1038/s41598-023-38675-9

**Published:** 2023-07-22

**Authors:** J. Jane Cao, Linghong Shen, James Nguyen, Kathleen Rapelje, Craig Porter, Evan Shlofmitz, Allen Jeremias, David J. Cohen, Ziad A. Ali, Richard Shlofmitz

**Affiliations:** 1 Department of Cardiology, St Francis Hospital and Heart Center, 100 Port Washington Blvd, Roslyn, NY USA; 2 Department of Cardiology, Shanghai Chest Hospital, Shanghai Jiaotong University School of Medicine, Shanghai, China

**Keywords:** Cardiology, Interventional cardiology

## Abstract

Plaques identified by Coronary CT angiography (CCTA) are important in clinical diagnosis and primary prevention. High-risk plaque features by CCTA have been extensively validated using optical coherence tomography (OCT). However, since their general diagnostic performance and limitations have not been fully investigated, we sought to compare CCTA with OCT among consecutive vessel sections. We retrospectively compared 188 consecutive plaques and 84 normal sections in 41 vessels from 40 consecutive patients referred for chest pain evaluation who had both CCTA and OCT with a median time lapse of 1 day. The distance to reference points were used to co-register between the modalities and the diagnostic performance of CCTA was evaluated against OCT. Plaque categories evaluated by CT were calcified, non-calcified and mixed. The diagnostic performance of CCTA was excellent for detecting any plaque identified by OCT with the sensitivity, specificity, negative and positive predictive values and accuracy of 92%, 98%, 99%, 84% and 93%, respectively. The lower than expected negative predictive value was due to failure of detecting sub-millimeter calcified (≤ 0.25 mm^2^) (N = 12) and non-calcified plaques (N = 4). Misclassification of plaque type accounted for majority of false negative findings (25/41, 61%) which was most prevalent among the mixed plaque (19/41, 46%). There was calcification within mixed plaques (N = 5) seen by CCTA but missed by OCT. Our findings suggest that CCTA is excellent at identifying coronary plaques except those sub-millimeter in size which likely represent very early atherosclerosis, although the clinical implication of very mild atherosclerosis is yet to be determined.

## Introduction

Coronary computed tomography angiography (CCTA) is a well-established modality for detecting coronary atherosclerosis. In addition, the findings of CCTA provide important diagnostic and prognostic information^1,2^. Emerging evidence also suggests that CCTA is promising in identifying plaque features that might be precursors of acute coronary syndrome (ACS) irrespective of coronary stenosis^3^. In fact, over 50% of adverse cardiac events occur in patients with non-obstructive coronary artery disease during follow up^1,3–5^. Optical Coherence Tomography (OCT) is an intravascular imaging modality that can accurately define plaque composition owing to its superior spatial resolution of 10 μm or less^6,7^. There have been a number of studies published comparing high-risk plaque features by CCTA with OCT^8–13^. However, the general diagnostic performance of detecting and differentiating coronary plaques by CCTA has not been fully investigated when using OCT as a reference. In this study, we sought to examine the diagnostic accuracy and limitations of CCTA by comparing consecutive coronary plaques identified by OCT to those by CCTA.

## Methods

We retrospectively reviewed 42 consecutive cases referred for chest pain evaluation where CCTA was followed by OCT within a median time of 1 day (interquartile range 6 days). All patients presented with stable or chronic coronary artery disease. The decision to perform coronary catheterization following CCTA and to use OCT was at the discretion of the attending physicians. The majority of coronary interventions at our institution were guided by intracoronary imaging with a large proportion guided by OCT. Patients' demographic information was captured prospectively in our CCTA database.

### Coronary CT angiogram

CCTA was performed on a 64-row detector scanner (Siemens Sensation Cardiac 64 or GE Lightspeed VCT 64). Images were acquired with electrocardiogram gating and bolus tracking. On average, 65–85 mL iodixanol contrast (Visipaque 320, GE Healthcare, Shanghai, China) was administered using a power injector at an infusion rate of 6 mL/s. Detector collimation was 64 × 0.625 mm, gantry rotation speed 330 ms/rotation, pitch 1.375, and tube voltage 120 kVp with a variable tube current set at a quality reference of 600 mAs. The adaptive iterative reconstruction was implemented and set at 40%. The reconstructions were performed with a kernel generating 0.625-mm-thick slices with an average field of view of 300–360 mm centered at the heart. The images were analyzed using a Vitrea workstation (Cannon Vital Images, MN, USA).

### Optical coherence tomography

OCT was performed using the Optis Dragonfly catheter (Abbott, Santa Clara, CA, USA) with longitudinal coverage of 75 mm. The segment of OCT imaging was confirmed using the angiographic co-registration feature of the OCT software. Once the catheter was in place, a contrast flush was injected and OCT image acquired via an automated pullback. Image quality was evaluated for blood swirl, chatter, and adequate distal and proximal ends prior to ending the study. OCT images were analyzed offline on an Abbott workstation.

### Image analysis

Of the 42 cases, 2 were excluded due to inadequate OCT image quality. Among 40 patients included in the analysis 1 had two vessels imaged by OCT. We evaluated the entire vessel segment available from OCT imaging with comparisons made with CCTA section by section contiguously. The plaque locations were matched between the two modalities using distances to fixed reference points such as side branches. In addition, the plaque in the vicinity was also an important landmark to match. To analyze the plaque, images based on multiplanar reformation and curved planar reformation were used. The vessel section was first defined as having or not having atherosclerotic plaque. If a plaque was present, the plaque type was further characterized by CCTA and OCT. Plaques defined on CCTA were based on the Society of Cardiac Computed Tomography^14^ guidelines and included calcified, non-calcified and mixed plaque composition. The non-calcified plaque was defined as discrete low attenuation surrounding vessel wall with well-defined border. Plaque features by OCT were defined as calcific, lipidic, fibro or fibro-lipidic, and mixed which included fibro-calcific, lipo-calcific, fibro-lipo-calcific^15^. For the purpose of comparison, fibrotic, lipidic, and fibro-lipidic were grouped as non-calcified plaque. The analysis was performed for the entire length of OCT image section by section consecutively. The plaques identified by OCT or normal section randomly selected on OCT were then matched to CT. The intimal thickening seen on OCT was not classified as having a plaque.

Two randomly selected sections without plaque were chosen from each vessel analyzed by OCT which were then matched on CCTA. In addition to classification as false positive and/or false negative by CCTA, discordant findings were further graded as misclassification, whereby plaque was present but the plaque type was not appropriately matched. For example, a mixed plaque by OCT that was seen as a calcified plaque by CCTA was considered misclassified. To compare the overall diagnostic performance, the detection of any plaque by CCTA was defined as a concordant finding. The images were initially analyzed by two cardiologists who were reading CCTA and OCT simultaneously to ensure the coregistrations of area of interests. Afterwards, CCTA and OCT were read again by independent readers who were blinded to the findings of the comparing modality.

### Statistical analysis

Continuous variables were described as mean ± standard deviation. Categorical variables were presented as percentage. Sensitivity, specificity, and positive and negative predictive values of CCTA to detect plaques and plaque features were determined using OCT findings as the reference standard.

### Ethics approval and consent to participate

All research was performed in accordance with the Declaration of Helsinki. This study was approved by the St. Francis Hospital Institutional Review Board. Informed consent was waived by the St. Francis Hospital Institutional Review Board.

## Results

There were 40 patients included in this study with an average age of 60 ± 10 years, 26% female. The cardiovascular risk factors were prevalent including 63% (N = 25) hypertension, 18% (N = 7) diabetes, 60% (N = 24) hyperlipidemia, and 33% (N = 13) smoking history.

**Figure 1:**
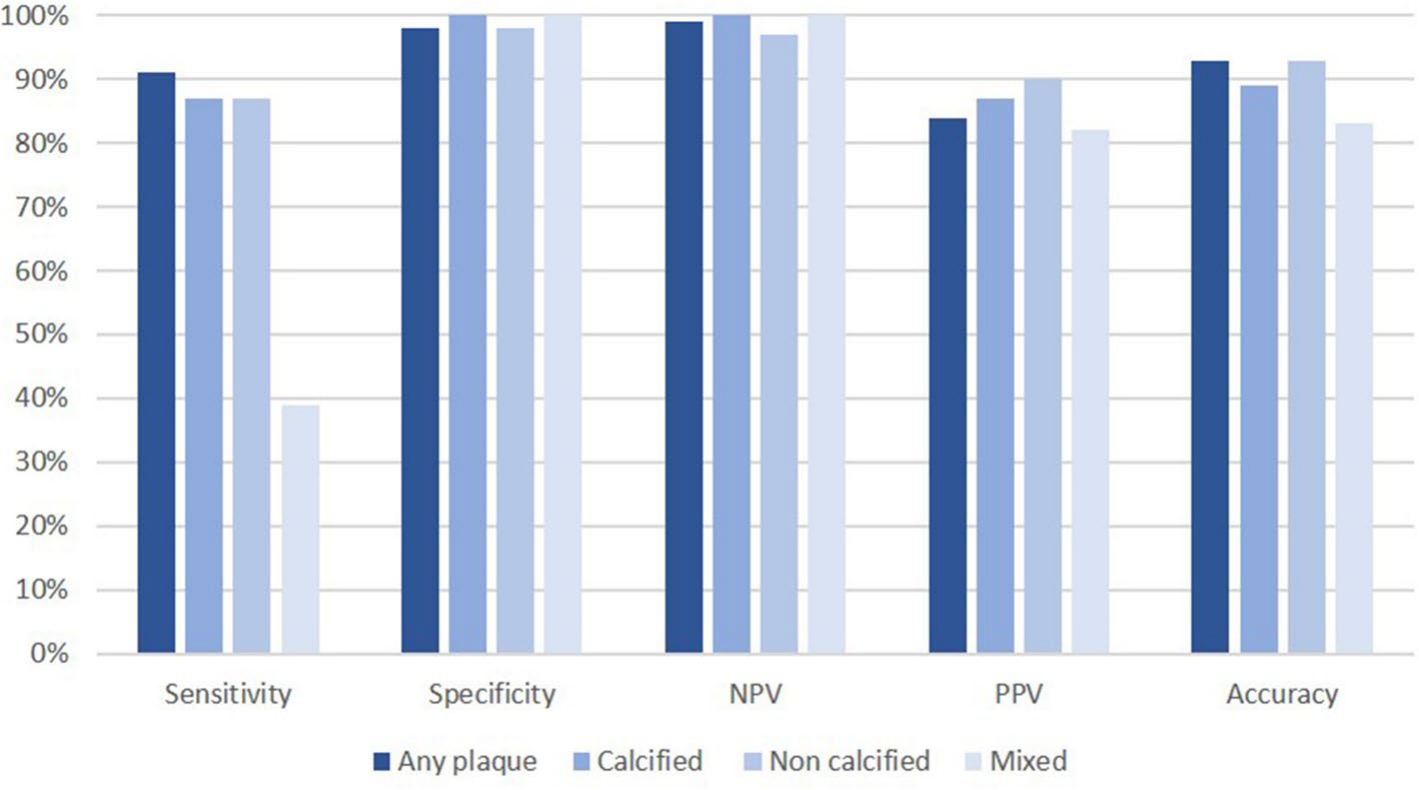
The sensitivity, specificity, positive predictive value (PPV), negative predictive value (NPV) and accuracy of coronary CT angiography in detecting plaque and plaque types compared with reference images from optical coherence tomography.

Of the 41 vessels analyzed, 24 were left anterior descending artery, 8 left circumflex or obtuse marginal, and 9 right coronary artery. Plaques extended from left main to left anterior descending coronary artery were included in the latter vessel category. Among 272 coronary vessel sections evaluated, CCTA was able to correctly identify 254 (93%) sections, either as having no plaque (82 out of 84) or having plaque (172 out of 188). The overall sensitivity, specificity, positive and negative predictive values and accuracy for detecting any plaque were 91%, 98%, 99%, 84% and 93%, respectively (Fig. 1).

We next evaluated CCTA’s ability to delineate plaque types. The concordance was 78% (147/188) with matching plaque types between CCTA and OCT. The discordance was 22% (41/188) summarized in Table 1 with majority due to misclassification of plaque types (31/43) especially the mixed plaque (19/43). There were 16 false negative plaques, where 12 calcified and 4 non-calcified plaques were seen on OCT but not on CCTA. In addition, there were 2 false positive cases where CCTA detected non-calcified plaques out of 84 sections without plaque by OCT.

**Table 1:** Comparisons of plaque features by coronary computed tomography angiography (CCTA) with reference images by optical coherence tomography (OCT).

Plaque features	By OCT	True positive by CT	False positive	False negative	Misclassification of plaque feature*
Calcific	87	74	0	13	1
Lipidic	35	30	0	5	2
Fibro- or fibro-lipidic	35	31	0	4	3
Mixed	31	12	0	19	19
Total plaques	188	147	0	41	25

*Misclassification of the plaque features was the cause of false negative finding.

**Figure 2:**
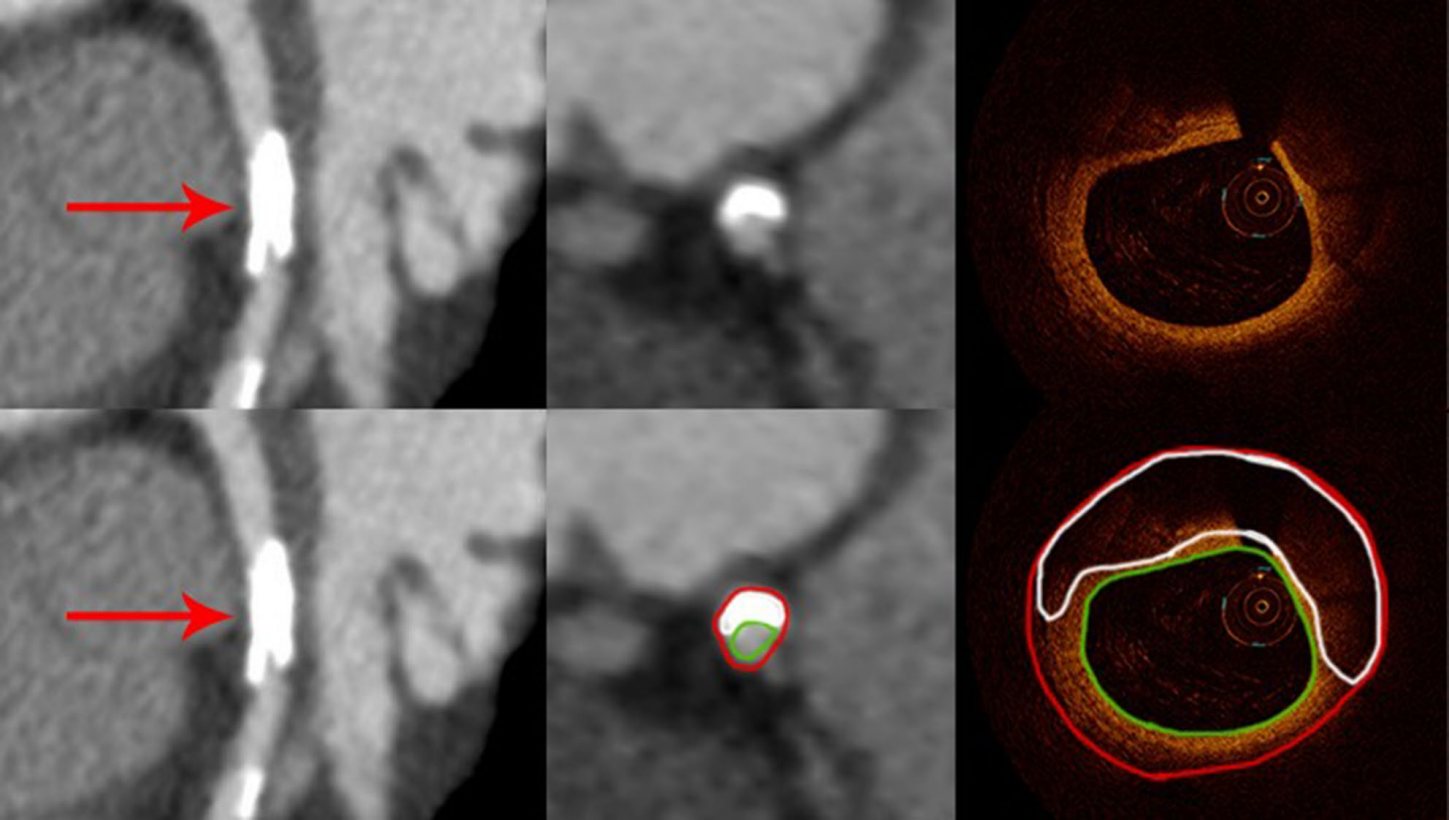
Curved multiplanar reformation computed tomographic images (left) and cross-sectional (middle) views of the left anterior descending artery from CCTA (red arrows) show a densely calcified plaque with hyperattenuated signal. The corresponding OCT image (right) demonstrates a thick calcification with a distribution similar to that on CT. The red contour encompasses the interpolated vessel wall and the green outline the vessel lumen.

**Figure 3:**
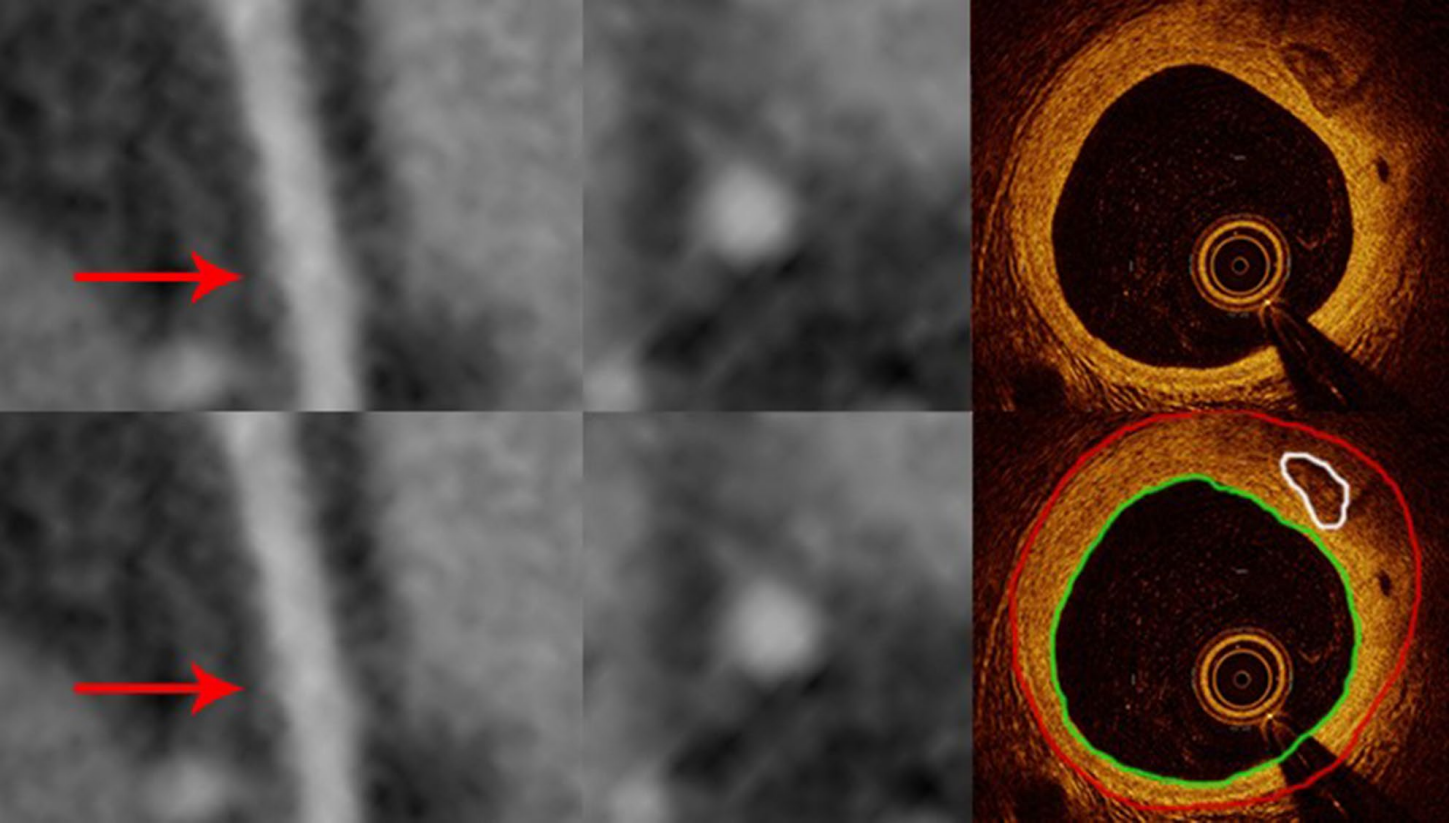
Curved multiplanar reformation computed tomographic images (left) and the cross-sectional (middle) views of the left anterior descending coronary artery from CCTA (red arrows) do not show coronary calcification seen on the corresponding OCT image (right) where a small calcified plaque of 0.25 mm^2^ is present. The red contours encompass the vessel wall and the green ones outline the vessel lumen.

Among 87 calcified plaques visualized by OCT, 74 (85%) were identified by CCTA (Fig. 2) and 12 were not identified due to small plaque size (≤ 0.25 mm^2^) (Fig. 3). The sensitivity, specificity, positive and negative predictive values, and accuracy for detecting calcified plaque by CCTA were 87%, 100%, 100%, 87% and 89% respectively.

**Figure 4:**
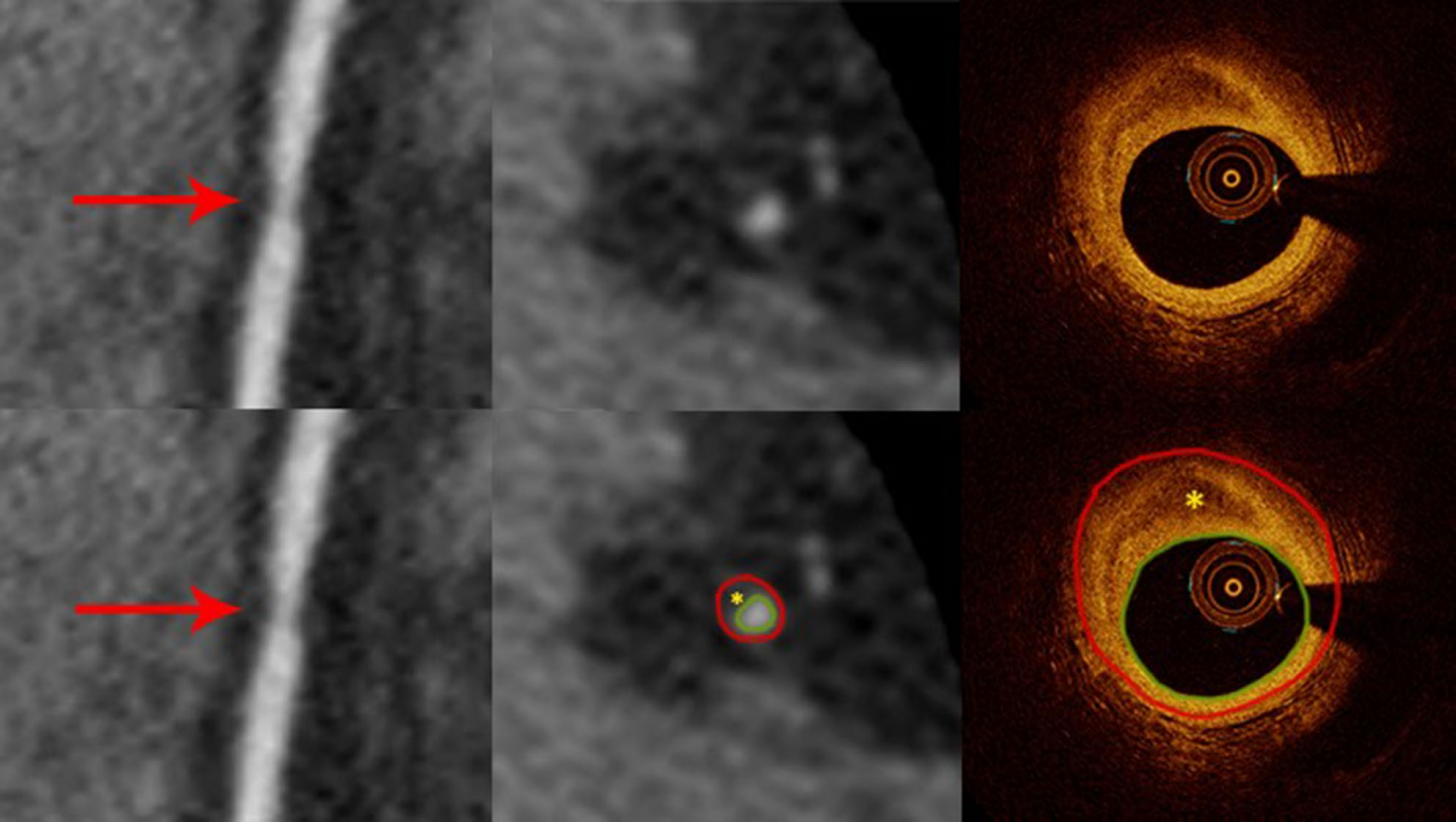
Curved multiplanar reformation computed tomographic images (left) and the cross-sectional (middle) views of the right coronary artery from CCTA (red arrows) reveals a hypoattenuated plaque where the vessel lumen is narrowed. The corresponding OCT image (right) shows a predominantly fibrotic plaque with a lipid pool (yellow asterisks). The red contours encompass the vessel wall and the green ones outline the vessel lumen.

There were 70 non-calcified plaques, either lipidic, fibrotic or fibro-lipidic by OCT. CCTA correctly identified 61 (87%) of them. The examples of non-calcified plaque by CCTA and OCT are shown in Fig. 4. Among 9 false negative cases 5 were mixed plaques seen on CT but classified as lipidic only (N = 4) or fibrotic only (N = 1) on OCT where calcification was not detectable. The sensitivity, specificity, positive and negative predictive values, and accuracy of CCTA was 87%, 98%, 97%, 90% and 93%, respectively. The lipidic plaque on OCT was associated with plaque signal intensity of 53 ± 30 Hounsfield units (HU) on CCTA. The signal intensity was higher for fibro-lipidic and fibrotic plaque of 77 ± 44 HU and 108 ± 53 HU, respectively.

**Figure 5:**
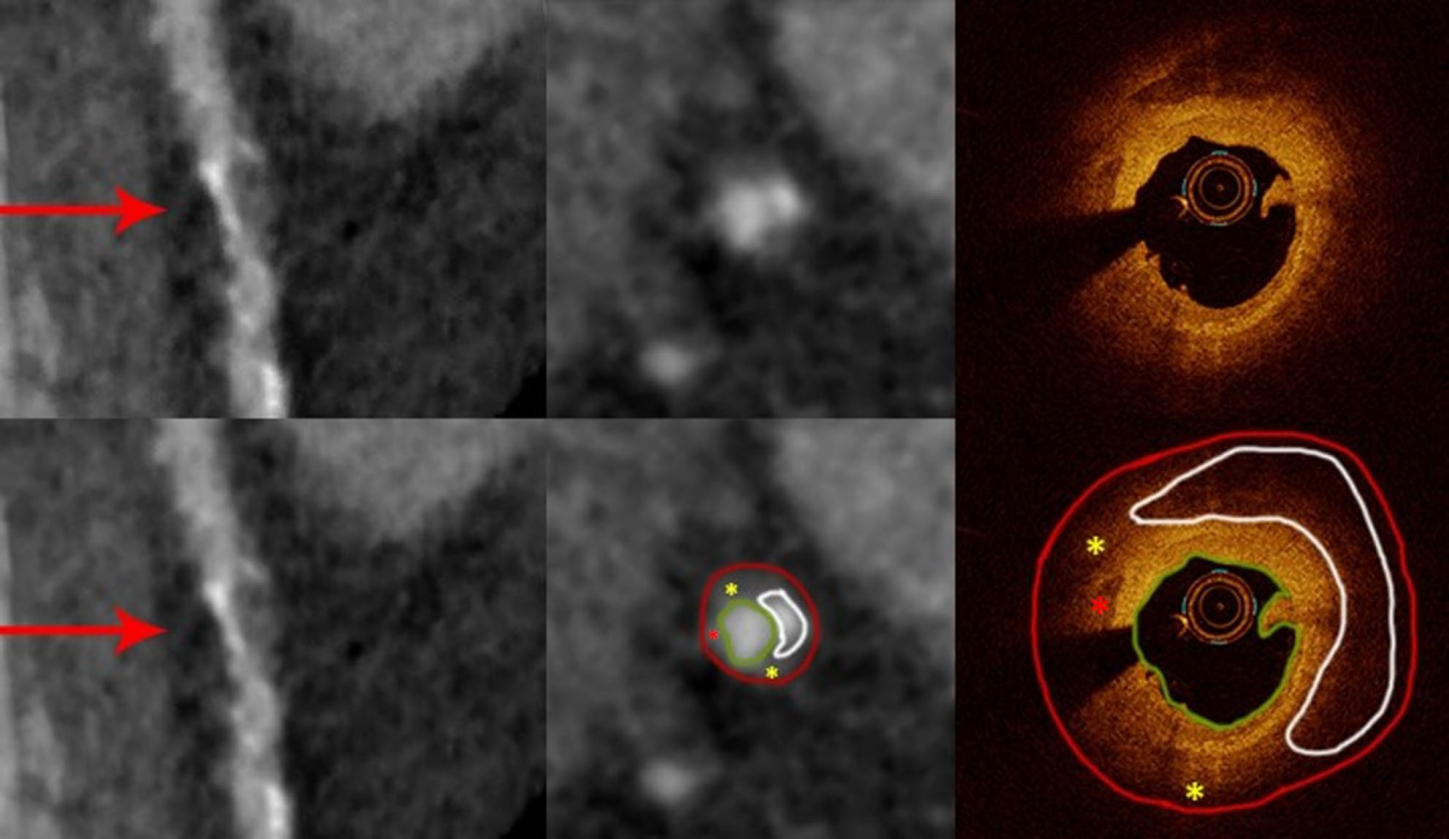
Curved multiplanar reformation computed tomographic images cross-sectional (middle) views of the left anterior descending artery from CCTA (red arrows) demonstrate a mixed plaque with hyperattenuated signal representing the calcified lesion and hypoattenuated signal representing the non-calcified lesion. OCT of the corresponding area shows luminal irregularity and thrombus, with 180 degree of calcium (white outline) with an additional small calcification (red asterisk), and surrounding areas of fibro-lipid plaque (yellow asterisks). The annotations on OCT are applied to the CCTA cross-sectional view (lower middle) where the calcified lesion (white outline) with an additional small calcification (red asterisk), and the surrounding area of non-calcified lesion (yellow asterisks) are matched (lower middle). The red contours encompass the interpolated vessel wall and the green ones outline the vessel lumen.

**Figure 6:**
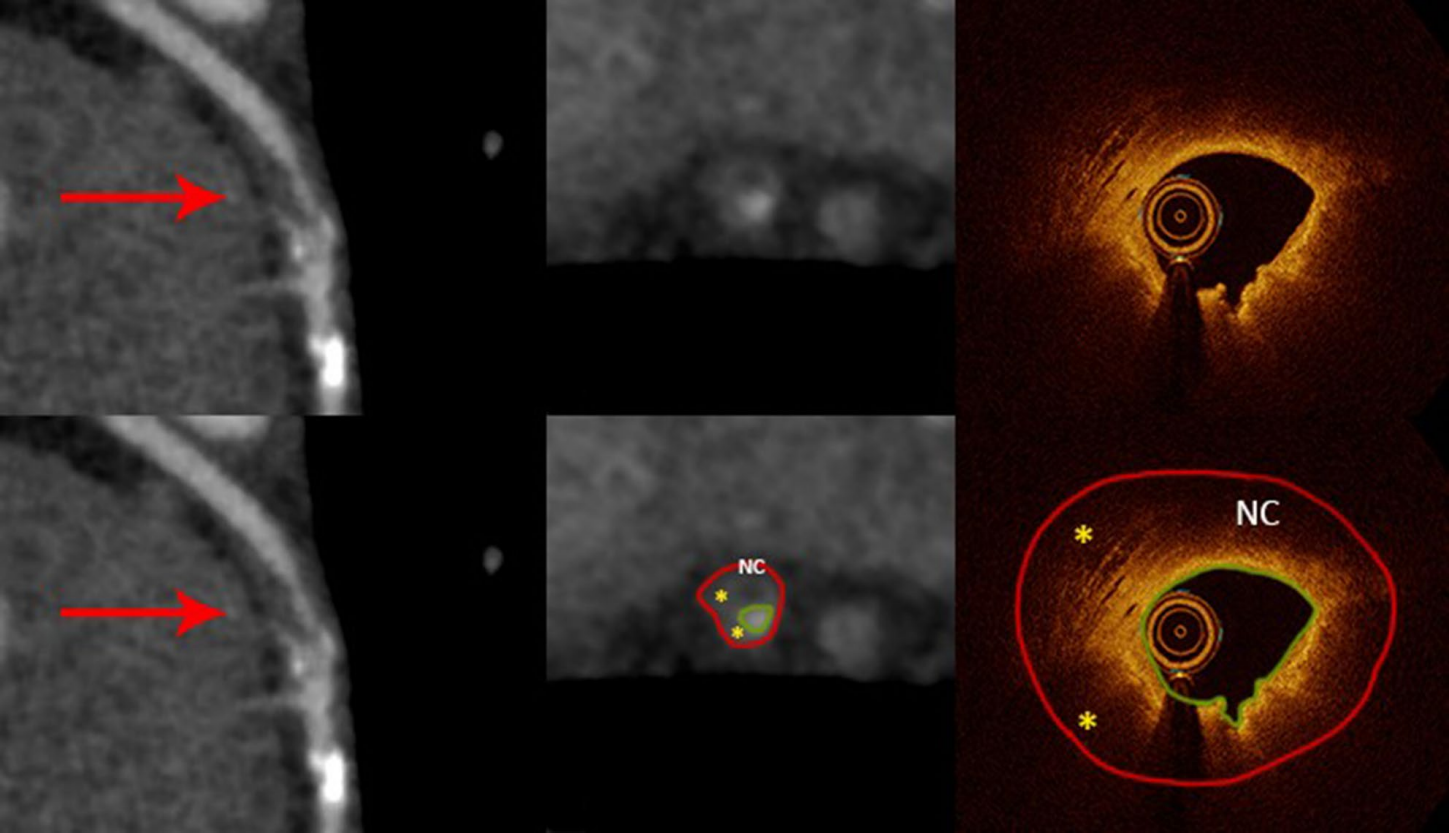
Curved multiplanar reformation computed tomographic images (left) and cross-section (middle) views of the left anterior descending artery from CCTA (red arrows) demonstrates hypoattenuated lesions with a dark core (yellow asterisks) giving rise to a ‘napkin ring’ appearance. The corresponding OCT image (right) reveals luminal irregularity with fibrofatty plaque (yellow asterisks) and a necrotic core (NC), which appears to match the dark core on CCTA (lower middle). The red contours encompass the vessel wall and the green ones outline the vessel lumen.

Among 31 mixed plaques identified by OCT 12 were correctly characterized by CCTA (Figs. 5 and 6). The remainder of 19 plaques were misclassified either as calcified only (N = 9) or non-calcified only (N = 10) by CCTA rendering the sensitivity, specificity, positive and negative predictive values, and accuracy of 39%, 100%, 100%, 82% and 83%, respectively.

## Discussion

In this study we demonstrated excellent diagnostic performance of CCTA in identifying coronary plaque and differentiating plaque types in comparison with OCT when analyzing consecutive plaques. However, CCTA is limited in detecting sub-millimeter calcified or non-calcified plaques likely due to lower spatial resolution relative to OCT, thereby compromising the negative predictive value of CCTA.

There have been a number of publications comparing the diagnostic performance of CCTA with OCT^8,10–13^. However, the focus of those studies is largely on high-risk plaques or plaque features of culprit lesion during ACS such as positive remodeling, low attenuation, spotty calcification, and napkin ring sign by CCTA^5^. Despite the higher prevalence seen in patients with ACS, those high-risk features seem to only account for a small number of cases with future ACS in the ICONIC trial^16^. To date, few study if any have investigated the performance of CCTA in detecting and delineating coronary plaques using OCT as reference standard. Nevertheless, such knowledge is highly relevant as we have now entered an era when atherosclerotic changes identified by CCTA are increasingly used not only to make diagnoses of coronary stenosis but also to guide therapy for primary prevention since such strategies have been shown to provide significant benefits of morbidity and mortality prevention in clinical trial^17^. In this study we compared CCTA with OCT in consecutive vessel sections to assess plaque of all types and sizes and found CCTA is excellent at detecting any coronary plaque with 93% accuracy. While it is expected that CCTA may miss very small plaques that are seen on OCT due to their 50-fold difference in spatial resolution, it is unclear which of the plaque types and their frequencies are undetectable by CCTA. In our study nearly 9% (16 out of 188) of all plaques were not detectable due to small size (≤ 0.25 mm^2^) which contributed to a lower than expected negative predictive value of CCTA than previously reported when using ex vivo specimens as reference^18,^ although tissue distortion or degradation is common in ex vivo tissues which may compromise the accuracy of plaque detection. We believe that these small plaques likely represent the beginning or early phase of coronary atherosclerosis though the clinical significance of sub-millimeter plaque is unknown when found in isolation.

The non-detectable small calcifications are not uncommon in our study accounting for nearly 14% of all calcified plaques. The ramifications of such small plaques may be inconsequential as subjects with zero coronary calcium score are generally associated with good outcomes. Nevertheless, the incidence of ACS is not zero and the non-detectable spotty calcification is potentially an unrecognized etiology^19,20^ adding to the list of established ones such as rupture of non-calcified plaque and thromboembolic risk.

While coronary plaque detection by CCTA is excellent, the delineation of specific plaque tissue type remains challenging largely owing to the relatively inferior spatial resolution of 0.6 mm when compared with OCT of 0.01 mm. In addition, calcium blooming from CT may further compromise the distinction between non-calcified and calcified compositions. Despite the large difference in spatial resolution, CCTA is highly specific in defining all three major plaque types, making it invaluable in non-invasive coronary plaque characterization, which is important for risk stratification^5,21–23^. Among all plaque types the low attenuation plaque, commonly associated with lipid-rich necrotic core, is most significantly associated with future risk of acute coronary syndrome^16,24,25^. Furthermore, the risk seems to be higher when the plaque burden is larger. To date, many physicians initiate primary prevention therapy such as statin in patients who are identified as having coronary atherosclerosis by CCTA, which has resulted in much reduced cardiovascular morbidity and mortality^22^. It is foreseeable that plaque features by CCTA may become the therapeutic targets, and the change of plaque characteristics may serve as a surrogate for treatment efficacy. To that end, it is critical to understand the accuracy of plaque evaluation by CCTA.

The near histological resolution of OCT^6,7,26,27^ allows for detection of very early coronary atherosclerosis in the form of tiny calcification or non-calcified plaque that is beyond the technical limits of CCTA. On the other hand, OCT has its limitations too^28^. In our study 7% (5 out of 70) of non-calcified plaque was characterized incorrectly by OCT due to failure to detect calcification seen on CCTA. This is an inherent limitation of OCT where relatively poor depth penetration prevents visualization of deep calcium^29^. In addition, superficial lipid accumulation can mask underlying calcification due to attenuation of light signals^30^. Nevertheless, OCT not only matches CT on detecting all the isolated calcified plaques but also identifies additional small calcification of sub-millimeter in size.

We recognize the limitations of our study. While OCT is an excellent reference standard owing to its near histological resolution it clearly has its limitations in the evaluation of calcification in mixed plaque, although OCT appears to be highly accurate in detecting isolated calcified plaque. While all of the CCTA and OCT evaluations were performed within days they did not occur on the same day. However, it is unlikely to expect significant change in plaque features within a short duration in the absence of ACS. The OCT pull back distance is typically 75 mm which limits the analyzable vessel length. So the analysis of CCTA is confined to the segments that are visualized by OCT. The current study was intended to characterize the basic plaque compositions in the categories of calcified, non-calcified and mixed plaques. Therefore, we did not systematically evaluate high-risk plaque features that have been reported previously. The negative predictive value of CCTA is likely much higher in the general population than reported in the present study due to the high prevalence of coronary atherosclerosis since only cases with advanced atherosclerosis on CCTA are likely to be selected by attending physicians to undergo OCT evaluation. In addition, The CCTA image quality overall is good despite using a 64-row CT scanner. Newer CT technology with higher spatial and temporal resolution may further improve the diagnostic accuracy of CCTA. Our sample size is relatively small which may limit the generalizability. Future larger studies may allow more comprehensive subgroup analyses for each plaque type and more importantly provide prognostication based on accurate plaque characterization.

To conclude, CCTA is excellent at characterizing coronary plaques when compared with OCT, but it may miss very early atherosclerosis although the clinical implications of very mild atherosclerosis are yet to be determined.

## Data Availability

The datasets used and/or analyzed during the current study are available from the corresponding author on reasonable request.
